# Cardiovascular diseases-related *GNB3* C825T polymorphism has a significant sex-specific effect on serum soluble E-selectin levels

**DOI:** 10.1186/s12950-016-0146-z

**Published:** 2016-12-09

**Authors:** Kokoè Mélinda Gbadoe, Nazha Berdouzi, Alex-Ander Aldasoro Aguiñano, Ndeye Coumba Ndiaye, Sophie Visvikis-Siest

**Affiliations:** UMR INSERM U1122; IGE-PCV “Interaction Gène-Environnement en Physiopathologie CardioVasculaire”, Faculté de Pharmacie, Université de Lorraine, Nancy, F-54000 France

**Keywords:** Sex-specific effect, Cell adhesion molecules, Selectins, *GNB3* gene, Cardiovascular diseases

## Abstract

**Background:**

The C825T polymorphism (rs5443) of the Guanine Nucleotide-Binding protein subunit β3 (*GNB3*) gene has been associated with obesity, essential hypertension, atherosclerosis, coronary diseases, and cerebrovascular events, but with some sex-specific effects. Its association with inflammatory mediators such as cell adhesion molecules has not been studied, although they are heavily involved in cardiovascular diseases’ (CVDs) processes. The aim of our study was then to investigate a possible sex-specific effect of the *GNB3* C825T polymorphism on serum soluble cell adhesion molecules such as E, P and L-selectins (sE, sP and sL-selectins).

**Results:**

Participants were from the STANISLAS Family Study and were free of chronic disease as CVDs or cancer. We included in total 771 subjects aged 6 to 58 years (391 males (50.71%) and 380 females (49.29%)). No significant association of rs5443 was observed in the whole population with serum sE, sP and sL-selectins after adjusting for age, sex, body mass index, systolic blood pressure, anti-inflammatory drugs and hormonal drugs consumption. A significant interaction of rs5443 was observed with sex for sE-selectin (*p* < 0.001), but not for sP and sL-selectins. After adjusting for covariables, the T allele was significantly associated with an additive increase effect on serum sE-selectin levels in males (β = 5.03 ± 2.18; *p* = 0.020), while a significant additive decrease effect was observed in females (β =−4.46 ± 2.06; *p* = 0.030). These associations stayed significant after correction for multiple tests (*p* = 0.045 in males and in females). The additive phenotypic variance was 21.54% in males versus 1.91% in females.

**Conclusions:**

In our Caucasian population, the *GNB3* C825T polymorphism showed a significant sex-specific effect on serum sE-selectin levels, with a disadvantage for males, as increased sE-selectin levels has been associated with CVDs outcomes. The T allele has been previously associated with the same CVDs as increased sE-selectin, but more often in males. The link we observed between this polymorphism and E-selectin is then consistent with previous findings, and helps to better understand the deleterious effect of the *GNB3* 825 T allele on CVDs outcomes in males. We revealed in this study an important pathway through which the *GNB3* gene induces CVDs’ outcomes.

## Background

The Guanine Nucleotide-Binding protein subunit β3 (*GNB3*) gene is present on chromosome 12 at the location 12p13 and presents a polymorphism called rs5443, resulting from a cytosine (C) to a thymine (T) substitution at position 825 (C825T) located in exon 10 of the gene [1]. This polymorphism is involved in cardiovascular pathophysiological processes and cardiovascular diseases (CVDs). Indeed, it has been associated with obesity [2], higher blood pressure [3], hypertension [1, 4, 5], carotid atherosclerosis [6], incident cerebrovascular events [7] and coronary diseases [8], even though these effects have also been controverted in the literature [9, 10].

Some of these studies showed a sex-specific effect of this single nucleotide polymorphism (SNP). Brand et al. observed a higher blood pressure in T homozygotes males than in C allele male carriers, while no significant difference was observed in females [3]. Hengstenberg et al. also observed a significantly higher prevalence of arterial hypertension in TT genotype subjects as compared to the other genotypes, but this association was predominantly found in men [4]. Frey et al. observed that the TT genotype was a significant risk factor for fatal and non-fatal myocardial infarction, independently of other established cardiovascular risk factors at a population level, but only in males, while in females no significant association was observed [8]. All these elements encourage further investigations of a sex-specific effect of this polymorphism.

Furthermore, the association of the *GNB3* 825 T allele with lipids, body mass index (BMI) [11], and blood pressure [1, 3–5] have been studied but not with molecules involved in inflammatory pathways such as cell adhesion molecules (CAM), while their importance in cardiovascular processes is well known [11, 12].

In this perspective, the aim of our present study was to investigate a possible sex-specific effect of the *GNB3* C825T polymorphism on the serum soluble cell adhesion molecules E, P and L-selectins (sE, sP and sL-selectins).

## Methods

### Study participants

Participants are from the STANISLAS (Suivi Temporaire annuel Non Invasif de la Santé des Lorrains Assurés sociaux) Family Study (SFS), a 15-year longitudinal monocentric family survey. Individuals were invited to three quinquennial medical check-ups that were held in 1994–1995, 1998–2000, and 2000–2003 [13, 14]. The SFS includes Caucasian volunteers, frequenting the Centre for Preventive Medicine of Vandoeuvre-lès-Nancy (East of France). All the subjects recruited were free of chronic diseases as CVDs or Cancer. Data on sE, sP and sL-selectins have been collected for subjects present during the second quinquennial medical check-up which occurred in 1998–2000. Among the 2,532 subjects (754 families) present at this second recruitment, we included those who were genotyped for the SNP rs5443, and for which data on the following factors were available: age, sex, BMI, systolic blood pressure (SBP), and anti-inflammatory drugs consumption (nonsteroidal drugs and corticosteroids). In females, we added a supplementary criterion: the availability of data about hormonal drugs consumption including oral contraceptives and hormonal replacement therapy containing oestrogen or progesterone).

### Blood samples and data collection

Serum concentrations of sE, sP and sL-selectins were measured using commercially available enzyme-linked immunosorbent essay according to the manufacturer’s specifications (ELISA kits, R&D Systems, Abingdon, Oxon, UK). The intra- and inter-assay coefficients of variation were as follows: sE-selectin, 9.4 and 14.9% (sensitivity: 0.027 ng/mL, assay range: 0.1–8 ng/mL); sP-selectin, 5.8 and 7.0% (sensitivity: 0.5 ng/mL, assay range: 0.8–46 ng/mL); sL-selectin, 8.9 and 11.7% (sensitivity: 0.3 ng/mL, assay range: 1.0–58 ng/mL), respectively.

Frozen aliquots of serum were stored in the Biological Resources Centre (BRC) “Interactions Gène-Environnement en Physiopathologie CardioVasculaire” (IGE-PCV).

Information about drug consumption and personal medical history was collected using relevant questionnaires and procedures under the supervision of trained nurses.

Weight and height were measured while the participants were standing in light clothing without shoes and BMI was calculated as weight in kilograms divided by height in meters squared.

Blood pressure was measured under constant temperature (19 °C-21 °C) and standardized conditions (supine position) using a manual sphygmomanometer. The recorded values were the means of 3 readings on 20 min intervals.

### Genotyping

Genomic DNA was extracted from venous blood samples by the salting-out method [15]. Genotyping of the SNP rs5443 was part of a multilocus assay performed with an immobilized probe approach designed by Roche Molecular Systems, Pleasanton, California, USA [16]. Genotyping was validated by classical Polymerase Chain Reaction methodology [17] in 50 individuals.

### Statistical analysis

The Hardy-Weinberg Equilibrium (HWE) of our study population was verified by a Chi square test. For statistical analyses, we used parametric method. The normality of the distribution was tested by Kolmogorov-Smirnov test. We controlled variances homogeneity of the compared groups, by the test of Levene.

The three phenotypes (sE, sP and sL-selectins) were introduced in a genetic additive model as quantitative variables, in an observational transversal and analytical design. The SNP effect was tested by a linear regression model, using an ANalysis Of VAriance (ANOVA). Covariates introduced in the model for adjustment are age, sex, BMI, SBP, and anti-inflammatory drug consumption. Age, BMI and SBP were considered in the model in their quantitative form. In the model testing the SNP effect in females, we added “hormonal drug consumption” as a supplementary qualitative covariate. Familial correlations in our study population were accounted for by using a linear mixed effects model that uses a relationship coefficient matrix as within pedigree correlation structure.

We performed analyses with the R package GWAF (Genome-Wide Association/Interaction Analysis and Rare Variant analysis with Family Data) [18]. The type I error, alpha was fixed at 5%. The power of our study has been calculated *a posteriori* with the Quanto software (http://biostats.usc.edu/Quanto.html).

Firstly we tested the association of the SNP rs5443 with phenotypes in the whole population, and secondly we tested the SNP interaction with sex. If a significant interaction of the SNP with sex was observed for a phenotype, we then tested a new association of the SNP with this phenotype separately in males and in females. A False Discovery Rate (FDR) correction was done in this case, for the SNP associations’ *p*-values.

## Results and discussion

**Fig. 1:**
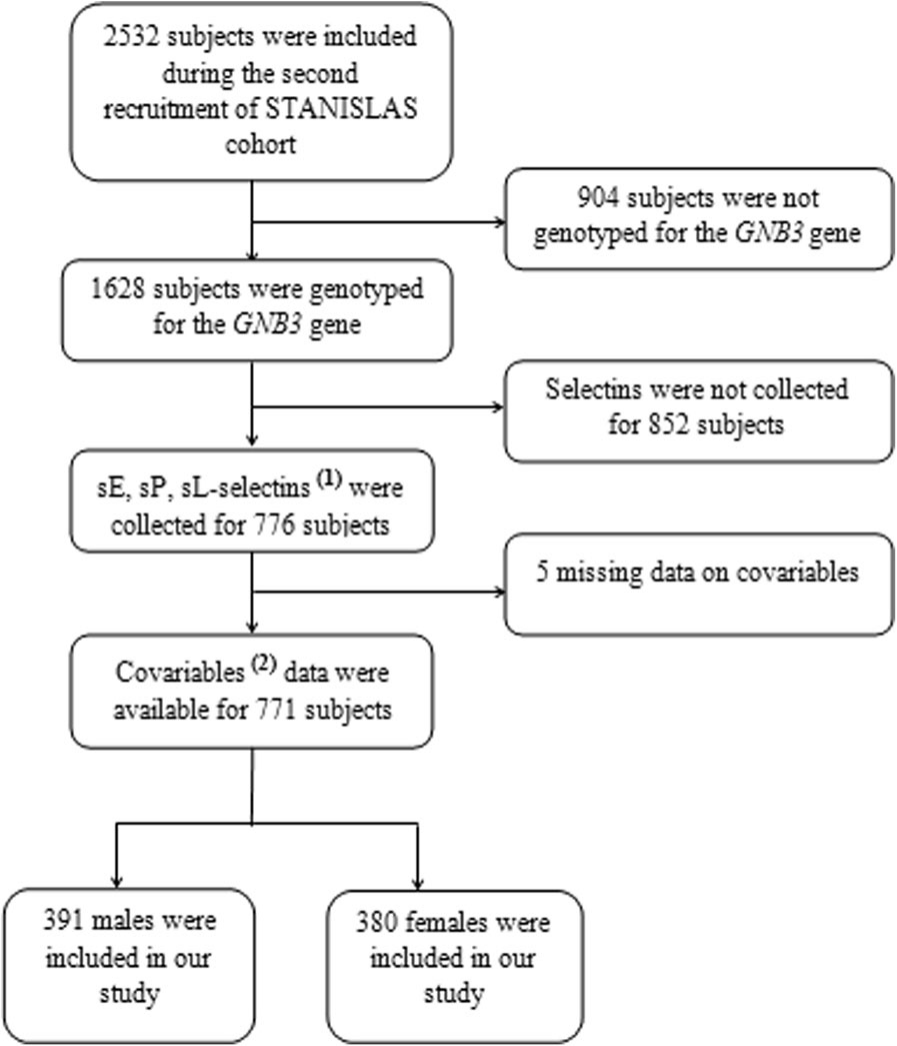
Flow chart of the selected population. 1. Serum Soluble E, P and L-selectins. 2. Age, sex, body mass index, systolic blood pressure, anti-inflammatory drugs, and hormonal drugs consumption (oral contraceptives and hormonal replacement therapy containing oestrogen or progesterone)

We included in total 771 subjects aged 6 to 58 years in our study, involving 391 males (50.7%) and 380 females (49.3%) (Fig. 1). Table 1 shows the characteristics of the study population, and Table 2, the genotypes distribution of the SNP rs5443 in the studied population. The total population showed no significant deviation from the HWE (*p* = 0.409). Table 3 shows the means and confidence intervals at 95% of quantitative variables introduced in the linear regression model testing the SNP effect on selectins.

**Table 1:** Characteristics of the study population

Population characteristics	Percentages (%)		
	Males *n* = 391	Females *n* = 380	Total population *N* = 771
BMI(kg/m^2^)			
< 18.5	11.5	13.7	12.6
18.5–25	61.6	66.3	63.9
25–30	22.5	15.5	19.1
> =30	4.3	17	4.4
Age (years)			
< 15	15.9	17.4	16.6
15–24	37.6	34.7	36.2
25–34	2.0	1.3	1.7
35–44	21.5	32.1	26.7
45–54	22.5	14.2	18.4
55–64	0.5	0.3	0.4
SBP(mmHg)			
< 120	41.7	62.6	52.0
120–139	50.9	31.6	41.4
140–159	7.4	5.0	6.2
> =160	0.0	0.8	0.4
Anti-inflammatory drug consumption			
Yes	3.8	5.0	4.4
No	96.2	95.0	95.6
Hormonal drug consumption			
Yes	0	31.6	15.6
No	100	68.4	84.4

*BMI* Body Mass Index, *SBP* Systolic Blood Pressure

**Table 2:** Genotype distribution and allele frequencies in the studied population

Genotypes	Males		Females		Total Population	
	n	%	n	%	n	%
CC	176	45.0	158	41.6	334	43.3
CT	171	43.7	168	44.2	339	44.0
TT	44	11.3	54	14.2	98	12.7
Total	391	100.0	380	100.0	771	100.0

Minor Allele Frequencies in males, Females and total population were respectively 33.5, 37.0, 35.7%

*p*-value of Hardy-Weinberg Equilibrium test was 0.4

**Table 3:** Means and confidence intervals of quantitative variables introduced in the linear regression model, studying the SNP rs5443 effect on serum soluble selectins in the STANISLAS population

Means CI (95%)			
Variables	Total population	Males	Females
sE-selectin (mg/l)	56.0 [54.0;58.0]	60.0 [57.1; 62.9]	51.8 [49.0; 54.5]
sP-selectin (mg/l)	132.5 [129.7; 135.3]	140.5 [136.4; 144.5]	124.4 [120.8; 128.0]
sL-Selectin (g/l)	1227.7 [1191.0; 1264.3]	1221.4 [1165.5; 1275.5]	1234.1 [1185.4; 1282.04]
Age (years)	29.4 [28.4; 30.4]	29.5 [28.1; 31.0]	29.3 [27.9; 30.7]
BMI (kg/m^2^)	22.5 [22.2; 22.8]	22.8 [22.4; 23.2]	22.2 [21.8; 22.6]
SBP (mmHg)	120.0 [119.1; 120.9]	122.4 [121.2; 123.6]	117.5 [116.3; 118.8]

CI(95%): Confidence Interval at 95%

Dependent variables: sE, sP, and sL-selectins (serum soluble E, P and L-selectins), Covariables: BMI, SBP, Age

*BMI* body mass index, *SBP* systolic blood pressure

### In the whole population

The additive model showed no significant result for sP, sL, and sE-selectins (respective *p*-values = 0.876, 0.158 and 0.770) after adjusting for covariables (sex, age, SBP, BMI, hormonal drugs, or anti-inflammatory drug consumption).

### Interaction of the SNP with sex

**Fig. 2:**
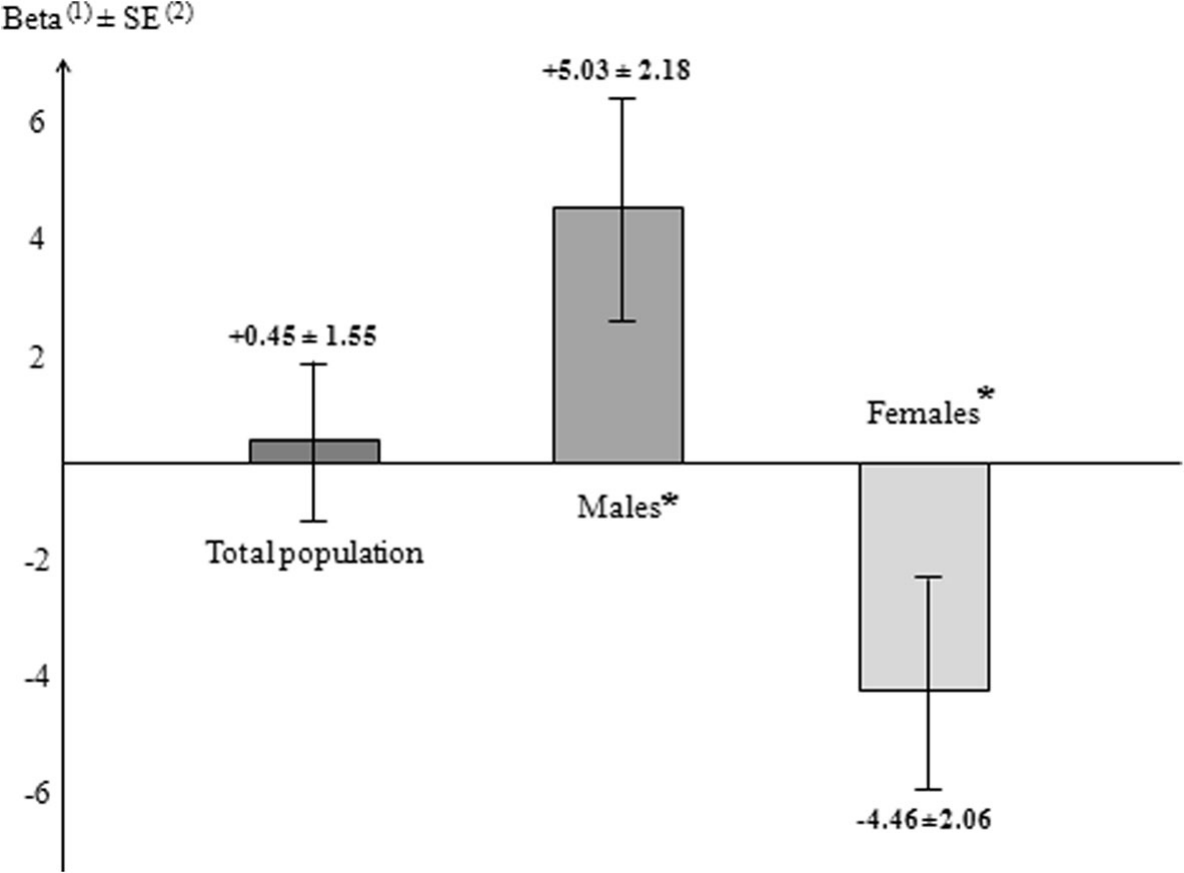
Effects of the *GNB3* 825 T allele on serum soluble E-selectin levels in the STANISLAS population. 1. The additive genetic effects of the *GNB3* 825 T allele on serum soluble E-selectins levels (mg/l), in the total population (*N* = 771), and separetly in males (*n* = 391) and females (*n* = 380). 2. Standard error of the effects. *P-value ≤ 0.05 (Significance level) after false discovery rate correction: The *GNB3* 825 T allele significantly increased serum soluble E-selectin levels in males and significantly decreased these levels in females, while in the total population (males + females), no significant effect was observed

After adjusting for covariables, the interaction of rs5443 with sex was significant with sE-selectin (*p* < 0.001) while the results with sP and sL-selectins were not significant (*p* = 0.079 and *p* = 0.607 respectively). Indeed, in the multivariate analysis, the T allele was significantly associated with an increased additive effect on serum sE-selectin levels in males (*p* = 0.020), while in females we observed a significantly decreased additive effect (*p* = 0.030). After correction by the FDR method, *p*-values remained statistically significant in males (*p* = 0.045) and females (*p* = 0.045). The effects of the T allele on sE-selectin in males, females and the whole population are illustrated in Fig. 2.

### Additive genetic variances

The proportion of sE-selectin variance explained by the genetic additive effect in males was 21.54% versus only 1.91% in females.

### Association of the C825T polymorphism with E-selectin

This study revealed a significant additive effect of the *GNB3* C825T polymorphism on serum sE-selectin levels, in males and females populations.

E-selectin also called Endothelial-Leucocyte Adhesion Molecule 1 (ELAM 1) is a cell surface glycoprotein expressed by endothelial cells (ECs) and is involved in leukocyte adhesion [19]. Its levels are increased during endothelial dysfunction and vascular remodelling [20, 21]. Increased levels of sE-selectin have been associated with atherosclerosis [22, 23], essential hypertension [24, 25], cerebrovascular diseases [26] and coronary artery diseases [27, 28]. The *GNB3* C825T polymorphism has been associated with these same CVDs, as aforementioned [1–8]. A link between the *GNB3* C825T polymorphism and E-selectin is then consistent with previous findings, even if current knowledge does not show a direct involvement of the *GNB3* gene in the production of E-selectin molecule. This molecule is rather encoded by the *SELE* (Selectin E) gene [29]. However, the *GNB3* gene may be involved in a mechanism regulating levels of E-selectin.

The *GNB3* gene encodes a beta subunit of the heterotrimeric guanine nucleotide-binding proteins also called G proteins which transmit signals from the cell surface to intracellular signal cascades [30]. It is established that G proteins play important roles in regulating the cardiovascular system [25]. Gudi et al. have studied the involvement of G proteins in mechanochemical signal transduction and reported that within the circulation, endothelial alignment, atherosclerotic lesion formation, vascular remodelling, and vasoactive molecule release are all mediated in part by hemodynamic forces, with G-protein activation [31]. Authors also concluded that G proteins are closely associated with the flow-stimulated mechanochemical transducer within endothelial cells. It has been demonstrated that fluid mechanical forces directly influence endothelial cell structure and function [32]. In this perspective, Chappel et al. showed that prolonged oscillatory fluid shear stress induces expression of endothelial cell leukocyte adhesion molecules, including E-selectin [33]. We suggest that the link between the *GNB3* C825T polymorphism and E-selectin found in this study is possible through G proteins’ effect within endothelial cells, by a mechanotransduction process. Otherwise, the *GNB3* C825T polymorphism is associated with increased transmembrane signal transduction [1]. In this SNP’ carriers, G proteins may therefore have enhanced activity within endothelial cells, and consequently generates high levels of E-selectin.

### A likely association of the *GNB3* C825T polymorphism with P-selectin

P-selectin is expressed in activated endothelial cells as well as E-selectin, while L-selectin is expressed on leukocytes [34]. Knowing the effect of G proteins within endothelial cells, we can assume that the *GNB3* C825T polymorphism association would also be positive for P-selectin, but not with L-selectin. Our results seem to follow this trend. Indeed, we found that the sex-specific effect of the SNP was likely with P-selectin (*p*-value = 0.08), but unlikely with L-selectin (*p*-value = 0.61). We think that in a larger population, the sex-specific effect will also be positive for P-selectin at a significance level of 5%. We specify that confounding factors as antiplatelet medications or anticoagulants can hide this association. In our study population, none of the subjects reported taking these drugs.

### Interaction of the SNP rs5443 with sex, on sE-selectin

In our study, increased levels of sE-selectin have been observed in males but not in females. We observed that the strength of the SNP effect on sE-selectin was approximately similar in males and in females (varying between 2.85–7.21 for males and 2.40–6.52 for females) while the direction of the effect was opposite in these two sex groups. We can then conclude on a significant qualitative interaction of this SNP with sex on sE-selectin levels (*p* < 0.001). This sex-specificity explains why we were not able to detect this SNP’s effect in the whole population, as the addition of the opposite effects resulted in an almost null global effect.

These results demonstrate the importance of looking for a sex-specific effect of one or more variants that can constitute a study in itself. Indeed, sex-specific studies can reveal interesting associations that would be hidden in classical genetic association studies.

In our study, we also observed that the additive variance explained by the SNP rs5443 was more than 20% in males while this proportion was ten times less in females. Genetic factors seem to have a more pronounced effect on E-selectin phenotype in males, while in females, other elements as constitutional or environmental factors and their interactions might be more implicated. We could explain this sex-specificity by (inter alia) hormonal interactions. Indeed, Caulin-Glaser et al., in their study on subjects with coronary artery disease, observed a statistically significant increase in sE-selectin levels in men and postmenopausal women not receiving oestrogen replacement therapy (ERT) compared with women receiving ERT [35]. Others studies revealed that, a decrease in the number of inflammatory cells is observed, in follicular phase, in cyclic women, when oestrogen level is high, compared to an increase in males and post-menopausal females [36, 37]. Then, the SNP interaction with sex in our study could be due to hormonal influence in females. Indeed, our study focused on a Caucasian population from an industrilized country and it has been reported that the age at menopause among women from this population is around midlife. [38]. The proportion of women over 50 years in our study is only 0.04% (17 women). Our results in females may therefore be influenced by this selection bias about hormonal status.

In males, a case-control study of Wang et al. showed a significant interaction between the *SELE* gene and sex on essential hypertension [39]. The *SELE* gene was not associated with essential hypertension in females. Knowing the effect of the *GNB3* 825 T allele on essential hypertension often found in males, the sex-specific effect of this polymorphism on E-selectin observed in our study is consistent with this finding.

Our results help to better understand in males, the deleterious effect of the *GNB3* gene on CVDs, as hypertension. We suggest that the *GNB3* C825T polymorphism may improve significantly endothelial cells activities in inflammatory pathways, by an enhanced mechanotransduction signalling in males carriers of this variant, and consequently induces susceptibility to CVDs.

### Limitations and perpectives

In our study, the significance regarding the effects of the 825 T allele on sE-selectin in males and females is marginal after FDR correction (*p* = 0.045). We acknowledge that a replication in a larger study population would ameliorate the data dispersion and significantly improve the precision of the estimates.

It would be interesting that replication studies test hormonal interaction with the *GNB3* C825T polymorphism on E-selectin. We were not able to test this interaction in our study, as the proportion of women over 50 years was low.

Although we have demonstrated in this study an effect of the *GNB3* C825T polymorphism on sE-selectin, the precise mechanism of this gene on E-selectin molecule need to be clarified by further investigations.

The results of our study support an involvement of the SNP rs5443 in CVDs, particularly in men. A question arising from this observation is whether special monitoring in males carrying this variant is needed, especially when the mean age of cardiovascular risk is reached. Controversial results of some studies on this issue did not allow authors to decide clearly about it. Otherwise, the impact of this variant on CVDs’ outcomes was investigated but it would be also interesting to focus on its impact as cardiovascular prognostic factor to determine whether the evolution of CVD in carriers of this polymorphism is adverse or not. This could lead to consider therapeutic targets fitting this profile of patients. In this context, knowledge about the mechanism of this variant in the cardiovascular pathophysiological process would be of great use.

## Conclusions

In this present study, we were able to highlight, in Caucasians, a sex-specific association between the *GNB3* 825 T allele and sE-selectin, with however a disadvantage for males concerning the known impact of increased E-selectin on CVDs’ outcomes. This association might be hidden in classical studies, because of the opposite effect observed in males as compared to females. Our findings revealed an important pathway through which the *GNB3* gene induces CVDs’ outcomes and help to better understand the deleterious effect of the 825 T allele on CVDs outcomes in males.

## Abbreviations

BMI: Body mass index
BRC IGE-PCV: Biological Resources Centre “Interactions Gène-Environnement en Physiopathologie CardioVasculaire”
CAM: Cell adhesion molecules
CVDs: Cardiovascular diseases
ERT: Oestrogen replacement therapy
FDR: False discovery rate
GNB3: Guanine nucleotide-binding protein subunit β3
INSERM: Institut National de la Santé et de la Recherche Médicale
SBP: Systolic blood pressure
SFS: STANISLAS (Suivi Temporaire Annuel Non-Invasif de la Santé des Lorrains Assurés Sociaux) Family Study
SNP: Single nucleotide polymorphism

## References

[1] Siffert W, Rosskopf D, Siffert G, Busch S, Moritz A, Erbel R, Sharma AM, Ritz E, Wichmann HE, Jakobs KH (1998). Association of a human G-protein beta3 subunit variant with hypertension. Nat Genet.

[2] Hwang IC, Kim KK, Ahn HY, Suh HS, Oh SW (2013). Effect of the G-protein beta3 subunit 825T allele on the change of body adiposity in obese female. Diabetes Obes Metab.

[3] Brand E, Wang JG, Herrmann SM, Staessen JA (2003). An epidemiological study of blood pressure and metabolic phenotypes in relation to the Gbeta3 C825T polymorphism. J Hypertens.

[4] Hengstenberg C, Schunkert H, Mayer B, Doring A, Lowel H, Hense HW, Fischer M, Riegger GA, Holmer SR (2001). Association between a polymorphism in the G protein beta3 subunit gene (GNB3) with arterial hypertension but not with myocardial infarction. Cardiovasc Res.

[5] Semplicini A, Grandi T, Sandona C, Cattelan A, Ceolotto G (2015). G-protein beta3-subunit gene C825T polymorphism and cardiovascular risk: an updated review. High Blood Press Cardiovasc Prev.

[6] Wascher TC, Paulweber B, Malaimare L, Stadlmayr A, Iglseder B, Schmoelzer I, Renner W (2003). Associations of a human G protein beta3 subunit dimorphism with insulin resistance and carotid atherosclerosis. Stroke.

[7] Casiglia E, Tikhonoff V, Boschetti G, Bascelli A, Saugo M, Guglielmi G, Caffi S, Rigoni G, Giordano N, Grasselli C (2012). The C825T GNB3 polymorphism, independent of blood pressure, predicts cerebrovascular risk at a population level. Am J Hypertens.

[8] Frey UH, Moebus S, Mohlenkamp S, Kalsch H, Bauer M, Lehmann N, Nothen M, Muhleisen TW, Stang A, Erbel R (2014). GNB3 gene 825 TT variant predicts hard coronary events in the population-based heinz nixdorf recall study. Atherosclerosis.

[9] Guo L, Zhang LL, Zheng B, Liu Y, Cao XJ, Pi Y, Li BH, Li JC (2013). The C825T polymorphism of the G-protein beta3 subunit gene and its association with hypertension and stroke: an updated meta-analysis. PloS one.

[10] Pereira TV, Kimura L, Suwazono Y, Nakagawa H, Daimon M, Oizumi T, Kayama T, Kato T, Li L, Chen S (2014). Multivariate meta-analysis of the association of G-protein beta 3 gene (GNB3) haplotypes with cardiovascular phenotypes. Mol Biol Rep.

[11] Ko KD, Kim KK, Suh HS, Hwang IC (2014). Associations between the GNB3 C825T polymorphism and obesity-related metabolic risk factors in Korean obese women. J Endocrinol Investig.

[12] Meng LL, Tang YZ, Ni CL, Yang M, Song HN, Wang G, Li YZ, Zhang M, Li DQ (2015). Impact of inflammatory markers on the relationship between sleep quality and incident cardiovascular events in type 2 diabetes. J Diabetes Complicat.

[13] Siest G, Visvikis S, Herbeth B, Gueguen R, Vincent-Viry M, Sass C, Beaud B, Lecomte E, Steinmetz J, Locuty J (1998). Objectives, design and recruitment of a familial and longitudinal cohort for studying gene-environment interactions in the field of cardiovascular risk: the Stanislas cohort. Clin Chem Lab Med.

[14] Visvikis-Siest S, Siest G (2008). The STANISLAS cohort: a 10-year follow-up of supposed healthy families. Gene-environment interactions, reference values and evaluation of biomarkers in prevention of cardiovascular diseases. Clin Chem Lab Med.

[15] Miller SA, Dykes DD, Polesky HF (1988). A simple salting out procedure for extracting DNA from human nucleated cells. Nucleic Acids Res.

[16] Cheng S, Grow MA, Pallaud C, Klitz W, Erlich HA, Visvikis S, Chen JJ, Pullinger CR, Malloy MJ, Siest G (1999). A multilocus genotyping assay for candidate markers of cardiovascular disease risk. Genome Res.

[17] Mullis KB, Faloona FA (1987). Specific synthesis of DNA in vitro via a polymerase-catalyzed chain reaction. Methods Enzymol.

[18] Chen MH, Yang Q (2010). GWAF: an R package for genome-wide association analyses with family data. Bioinformatics (Oxford, England).

[19] Bevilacqua MP, Pober JS, Mendrick DL, Cotran RS, Gimbrone MA (1987). Identification of an inducible endothelial-leukocyte adhesion molecule. Proc Natl Acad Sci U S A.

[20] Chae CU, Lee RT, Rifai N, Ridker PM (2001). Blood pressure and inflammation in apparently healthy men. Hypertension.

[21] Dorr O, Liebetrau C, Mollmann H, Gaede L, Troidl C, Rixe J, Hamm C, Nef H (2014). Soluble fms-like tyrosine kinase-1 and endothelial adhesion molecules (intercellular cell adhesion molecule-1 and vascular cell adhesion molecule-1) as predictive markers for blood pressure reduction after renal sympathetic denervation. Hypertension.

[22] Galkina E, Ley K (2007). Vascular adhesion molecules in atherosclerosis. Arterioscler Thromb Vasc Biol.

[23] Ma S, Tian XY, Zhang Y, Mu C, Shen H, Bismuth J, Pownall HJ, Huang Y, Wong WT (2016). E-selectin-targeting delivery of microRNAs by microparticles ameliorates endothelial inflammation and atherosclerosis. Sci Rep.

[24] Blann AD, Tse W, Maxwell SJ, Waite MA (1994). Increased levels of the soluble adhesion molecule E-selectin in essential hypertension. J Hypertens.

[25] De Caterina R, Ghiadoni L, Taddei S, Virdis A, Almerigogna F, Basta G, Lazzerini G, Bernini W, Salvetti A (2001). Soluble E-selectin in essential hypertension: a correlate of vascular structural changes. Am J Hypertens.

[26] Richard S, Lagerstedt L, Burkhard PR, Debouverie M, Turck N, Sanchez JC. E-selectin and vascular cell adhesion molecule-1 as biomarkers of 3-month outcome in cerebrovascular diseases. J Inflamm (London, England). 2015;12. 10.1186/s12950-015-0106-z PMC4634720 26543408

[27] Hwang SJ, Ballantyne CM, Sharrett AR, Smith LC, Davis CE, Gotto AM, Boerwinkle E (1997). Circulating adhesion molecules VCAM-1, ICAM-1, and E-selectin in carotid atherosclerosis and incident coronary heart disease cases: the Atherosclerosis Risk In Communities (ARIC) study. Circulation.

[28] Zakynthinos E, Pappa N (2009). Inflammatory biomarkers in coronary artery disease. J Cardiol.

[29] Collins T, Williams A, Johnston GI, Kim J, Eddy R, Shows T, Gimbrone MA, Bevilacqua MP (1991). Structure and chromosomal location of the gene for endothelial-leukocyte adhesion molecule 1. J Biol Chem.

[30] Elefsinioti AL, Bagos PG, Spyropoulos IC, Hamodrakas SJ (2004). A database for G proteins and their interaction with GPCRs. BMC Bioinformatics.

[31] Gudi SR, Clark CB, Frangos JA (1996). Fluid flow rapidly activates G proteins in human endothelial cells. Involvement of G proteins in mechanochemical signal transduction. Circ Res.

[32] Dewey CF, Bussolari SR, Gimbrone MA, Davies PF (1981). The dynamic response of vascular endothelial cells to fluid shear stress. J Biomech Eng.

[33] Chappell DC, Varner SE, Nerem RM, Medford RM, Alexander RW (1998). Oscillatory shear stress stimulates adhesion molecule expression in cultured human endothelium. Circ Res.

[34] Tedder TF, Steeber DA, Chen A, Engel P (1995). The selectins: vascular adhesion molecules. FASEB J.

[35] Caulin-Glaser T, Farrell WJ, Pfau SE, Zaret B, Bunger K, Setaro JF, Brennan JJ, Bender JR, Cleman MW, Cabin HS (1998). Modulation of circulating cellular adhesion molecules in postmenopausal women with coronary artery disease. J Am Coll Cardiol.

[36] Burger HG, Hale GE, Robertson DM, Dennerstein L (2007). A review of hormonal changes during the menopausal transition: focus on findings from the Melbourne women’s midlife health project. Hum Reprod Update.

[37] Verthelyi D (2001). Sex hormones as immunomodulators in health and disease. Int Immunopharmacol.

[38] Gold EB (2011). The timing of the age at which natural menopause occurs. Obstet Gynecol Clin N Am.

[39] Wang Z, Liu Y, Liu J, Liu K, Lou Y, Wen J, Niu Q, Wen S, Wu Z (2010). E-selectin gene polymorphisms are associated with essential hypertension: a case-control pilot study in a Chinese population. BMC Med Genet.

